# A systematic evaluation of text mining methods for short texts: Mapping individuals’ internal states from online posts

**DOI:** 10.3758/s13428-024-02381-9

**Published:** 2024-04-04

**Authors:** Ana Macanovic, Wojtek Przepiorka

**Affiliations:** Department of Sociology/ICS, Utrecht University, Utrecht, The Netherlands

**Keywords:** Text mining, Natural language processing, Machine learning, Large language models, Big data

## Abstract

Short texts generated by individuals in online environments can provide social and behavioral scientists with rich insights into these individuals’ internal states. Trained manual coders can reliably interpret expressions of such internal states in text. However, manual coding imposes restrictions on the number of texts that can be analyzed, limiting our ability to extract insights from large-scale textual data. We evaluate the performance of several automatic text analysis methods in approximating trained human coders’ evaluations across four coding tasks encompassing expressions of motives, norms, emotions, and stances. Our findings suggest that commonly used dictionaries, although performing well in identifying infrequent categories, generate false positives too frequently compared to other methods. We show that large language models trained on manually coded data yield the highest performance across all case studies. However, there are also instances where simpler methods show almost equal performance. Additionally, we evaluate the effectiveness of cutting-edge generative language models like GPT-4 in coding texts for internal states with the help of short instructions (so-called zero-shot classification). While promising, these models fall short of the performance of models trained on manually analyzed data. We discuss the strengths and weaknesses of various models and explore the trade-offs between model complexity and performance in different applications. Our work informs social and behavioral scientists of the challenges associated with text mining of large textual datasets, while providing best-practice recommendations.

## Introduction

The rise of big data has confronted social and behavioral scientists with extensive amounts of text, challenging their conventional methodological approaches (Goldberg, 2015; Lupyan & Goldstone, 2019; Salganik, 2017). Ordinary individuals interacting in online spaces generate a growing amount of textual information about themselves and the social world (Golder & Macy, 2014). These short online-generated texts stem from real-life behaviors and social interactions and can be collected unobtrusively and in real time (Golder & Macy, 2014; Rauthmann, 2020), allowing us to overcome some of the limitations of other data collection methods (Radford & Lazer, 2019). These texts serve as a valuable resource for inference on internal human states such as character traits, individual norms, attitudes, opinions, values, sentiments, and motives (Boyd et al., 2015; Boyd & Schwartz, 2021; Evans & Aceves, 2016; Hoover et al., 2020).

Drawing inferences on internal states from texts is, in many ways, analogous to capturing constructs using questionnaires or experimental designs (Kennedy et al., 2022). The fundamental assumption here is that internal states manifest in the way individuals use language. Past research has demonstrated that this assumption holds in many cases, with speech and writing reflecting personality traits, sociodemographic characteristics, personal values, and moral concerns (Boyd et al., 2015; Matsuo et al., 2019; Schultheiss, 2013; Schwartz et al., 2013; Tausczik & Pennebaker, 2010). Textual measures have mostly been validated against self-reports, showing different levels of correlation on a range of internal states (Boyd et al., 2015; Kennedy et al., 2021; Koutsoumpis et al., 2022; Lykousas et al., 2019; Malko et al., 2021; Matsuo et al., 2019; Mozes et al., 2021; Pellert et al., 2022).

Drawing inference from text, in general, hinges on another fundamental assumption: that the meaning conveyed in language can be interpreted and quantified (Ignatow, 2016). This assumption underlies the gold standard of quantitative text analysis in the social sciences (Iliev et al., 2015; Nelson et al., 2021; Tausczik & Pennebaker, 2010), which involves defining theoretical concepts of interest (Kennedy et al., 2022) prior to the selection of a textual corpus which will be analyzed with the help of multiple trained human coders (Krippendorff, 2004a). When the goal is inference on internal states, the assumption becomes more specific: external observers can use cues in verbal behavior to deduce individuals’ internal states (Koutsoumpis et al., 2022). On the one hand, understanding how certain states are reflected in language is a distinctly human ability (Iliev et al., 2015; Kennedy et al., 2022). On the other hand, psychological research has noted an asymmetry between how individuals perceive their own states and how external observers assess these very states (Vazire, 2010). This asymmetry can result in a higher correlation between textual features and observer reports than between textual features and self-perceptions of one’s traits and states (Koutsoumpis et al., 2022). Furthermore, certain internal states appear to be reflected more clearly in text than others (Kennedy et al., 2021). Evaluating the construct validity of measurements extracted from text and relating them to alternative forms of measurement are still ongoing tasks.

Even with these limitations in mind, using texts for inference of internal psychological states can prove useful in many applications (Dehghani et al., 2014; Hasan & Ng, 2014; Hoover et al., 2020; Kröll & Strohmaier, 2009; Liu et al., 2012; Pennebaker et al., 2015; Prabhakaran et al., 2012; Schultheiss, 2013; Tay et al., 2020). Although inference of human analysts is not without its shortcomings (Song et al., 2020), when best-practice advice is followed, it provides us with a largely valid and reliable means of measuring the presence of concepts of interest in texts (Krippendorff, 2004a). However, when working with a large number of texts, manual coding quickly becomes resource-intensive and time-consuming. Instead of disregarding textual data that cannot be analyzed manually, researchers can utilize text mining techniques to extend their manual analyses to the entire large corpus of texts (Iliev et al., 2015; Kennedy et al., 2022; Macanovic, 2022; Tausczik & Pennebaker, 2010).^1^

However, the proliferation and increasing availability of diverse text mining methods presents researchers with a number of challenging questions. Which method most accurately approximates human coding for a given task? Can established dictionary methods be relied upon, or should we invest time in understanding complex large language models? How much can we trust accessible new implementations that require minimal technical knowledge on the part of researchers? How do our decisions at various analytical stages affect the performance of different methods? Here, we aim to provide comprehensive answers to these questions. Building on past research tackling some of the questions above (Barberá et al., 2021; Kusen et al., 2017; van Atteveldt et al., 2021; Yadollahi et al., 2018), we conduct a systematic survey aimed at social and behavioral scientists interested in using text mining to reliably extend manual coding of texts for various internal states. We examine methods of varying degrees of complexity and consider the impact of different analytical choices. Further, we consider the trade-offs between complexity and transparency, and between convenience and performance. Our survey includes four applications of the inference of internal states from short texts with different levels of complexity.

We assess how several families of text mining methods perform against the gold standard of systematic coding carried out by trained human coders (Bonikowski & Nelson, 2022; Grimmer et al., 2022). We first consider a family of methods familiar to many researchers: dictionary methods that identify words relevant to the concept of interest in text. Here, we also present a novel approach for easy generation of custom dictionaries from manually coded data. Next, we discuss supervised machine learning classification methods that infer coding patterns from manually coded data and apply them to new texts. Finally, we explore the potential of the cutting-edge zero-shot classification approach which involves instructing large language models with simple text-based instructions for coding. While dictionary methods remain the preferred method in social and behavioral sciences, the latter approaches hold great potential for nuanced coding of internal states (Boyd & Schwartz, 2021; Rathje et al., 2023).

This paper is structured as follows: We first provide a brief outline of the analytical framework for automatic text analysis. Then, we discuss text preparation and describe various text mining methods. Finally, we evaluate the performance of automatic methods against the gold standard of human manual coding on four datasets from online platforms. The textual corpora in these datasets were, depending on their content, manually coded for motives, moral norms, emotional states, and hateful attitudes.

## Analytical framework for automatic text analysis

In this section, we outline the framework for automatic text analysis, consider the characteristics of different text mining methods, and discuss how their performance can be compared to human coding.

### Step 1: Manual coding

The first step includes defining concepts of interest and determining a corresponding coding scheme that helps seek them in text (Kennedy et al., 2022; Krippendorff, 2004a). Next, researchers need to select a corpus of texts they want to analyze,^2^ and sample a subset that will be manually coded (Figueroa et al., 2012; Wang et al., 2022).^3^ Manual coding can involve either a small group of expert coders (Krippendorff, 2004a) or a large number of less-trained crowd coders (Benoit et al., 2016; Marquardt et al., 2017). In either case, it is crucial that every text is independently coded by multiple coders (Barberá et al., 2021; Krippendorff, 2004a; Marquardt et al., 2017; van Atteveldt et al., 2021), with their agreement reported and held to a satisfactory standard (Krippendorff, 2004b; Song et al., 2020).^4^ Both the sampling decisions and the level of coder agreement can impact the performance of automatic text analysis methods (Wang et al., 2022).

### Step 2: Method choice

The next step is to choose a text mining method to analyze the whole textual corpus. We discuss several families of methods that can be used for this task.

#### Dictionary methods

Dictionary methods use word lists (dictionaries) to categorize texts. For instance, when identifying emotions in text, a dictionary would map words that correspond to each emotion, such as joy or surprise. If available, researchers can use pre-existing dictionaries (ready-made dictionaries [RMDs]), such as the widely used Linguistic Inquiry and Word Count (LIWC) dictionary (Pennebaker et al., 2015).

An alternative is to create a custom-made dictionary (CMD) suited for the particular coding scheme and dataset at hand (Bonikowski & Gidron, 2016; Macanovic, 2022; Nelson et al., 2021; Spörlein & Schlueter, 2021). We present a straightforward approach that uses manually coded data to automatically generate dictionaries using the concept of word keyness from computational linguistics (Gabrielatos, 2018). To illustrate this procedure, consider the following example: If, in our sample of 1,000 manually coded texts, we have coded 100 texts for the emotion category of “joy,” we denote these 100 texts as the “target group” and the remaining 900 texts as the “reference group”. For instance, if the word “happy” occurs 20 times in the target group, but only 10 times in the reference group, we use a word keyness measure to assess the extent of the difference in word occurrence between the groups. We test two approaches: one using the likelihood ratio measure of statistical significance and the other using the %DIFF measure capturing the effect size of the difference (Gabrielatos, 2018).^5^ If we determine that “happy” occurs significantly more often in the target group, we include it as a dictionary entry for the category of “joy.” This simple approach can further be extended using more sophisticated methods of word selection (King et al., 2017) and dictionary expansion (Di Natale & Garcia, 2023; Garten et al., 2018).

#### SML classification methods

Supervised machine learning (SML) classification methods use the manually coded sample as training data to identify patterns in text features (see Sect. "Step 3: Data representation") which correspond to different coding categories. These patterns are then used to automatically code new, unseen data (Aggarwal & Zhai, 2012; Jurafsky & Martin, 2009; Nelson et al., 2021). We first consider a very simple SML model—logistic regression—which calculates the significance of each textual feature in determining whether a text belongs to a category or not (Jurafsky & Martin, 2009).^6^

We then assess two more complex algorithms. The random forest (RF) algorithm relies on decision trees: algorithms that iteratively infer which words are helpful in distinguishing between different coding categories (Aggarwal & Zhai, 2012; Rhys, 2020). RF operates under the assumption that the collective performance of multiple decision trees surpasses individual trees’ performance and, thus, averages many individual tree outputs to determine words relevant for each coding category (Rhys, 2020). As some of the coding schemes for internal states allow for several categories to co-occur in texts (e.g., both joy and surprise), we also consider a so-called multi-label RF algorithm that accounts for such co-occurrences (Tsoumakas & Katakis, 2007).^7^ A support vector machine (SVM) algorithm represents texts in a multidimensional space and identifies a multidimensional surface, or hyperplane, which best separates texts into different categories based on the words they contain (Aggarwal & Zhai, 2012; Rhys, 2020).

Finally, we evaluate the possibility of using transfer learning, a procedure where large language models (LLMs) that were already trained to learn text representations from large textual datasets (see Section "Step 3: Data representation") are adapted in order to perform specific tasks (Chae & Davidson, 2023; Do et al., 2022). When neural networks such as LLMs are used for text classification, they can be thought of as stacked linear regressions which iteratively process textual features and dynamically learn the weights that determine how important each feature is for predicting each coding category (Jurafsky & Martin, 2021). The final layer of the model (the classification head) then uses a logistic function to transform these weights into a binary outcome denoting whether a text belongs to a category of interest or not. We evaluate BERT , RoBERTa, and BERTweet, powerful transformer LLMs that were pre-trained on large textual datasets. We perform fine-tuning, a procedure during which these models learn how to classify texts into categories of our interest based on manually coded examples. These models belong to the family of autoencoding transformer language models which are, in general, considered a suitable choice for text classification (Vaswani et al., 2018).

#### Zero-shot classification

More recently, certain generative LLMs have been noted to develop broad pattern recognition abilities during pre-training, which allows them to perform various tasks without any fine-tuning on manually coded data (Brown et al., 2020). Given the nature of the task, researchers have been using the category of autoregressive large language transformer models designed for text generation to perform zero-shot classification (Gilardi et al., 2023; Ollion et al., 2023). Although autoencoding models mentioned previously (e.g., BERT) appear to be more appropriate for classification (Minaee et al., 2021), here we also assess the performance of GPT-3.5-turbo and GPT-4, two powerful autoregressive generative models. Unlike the autoencoding models for classification that calculate the probabilities of texts belonging to each coding category, generative models generate the coding category for each text when provided with a natural language instruction (i.e., a prompt).

Autoregressive generative models can, in general, be instructed by providing them with (1) a larger number of manually coded examples (fine-tuning, as discussed with regard to autoencoding models), (2) a few representative examples (i.e., few-shot learning), or (3) only coding instructions in natural language (i.e., zero-shot learning) (Liu et al., 2021). Recent work has highlighted, in particular, the good performance of zero-shot classification on some tasks  (Brown et al., 2020; Chae & Davidson, 2023), suggesting that this approach could support social scientific inquiry while reducing the costs and effort associated with fine-tuning of large language models (Rathje et al., 2023). This is why we focus on zero-shot classification capabilities of generative models.

### Step 3: Data representation

**Fig. 1:**
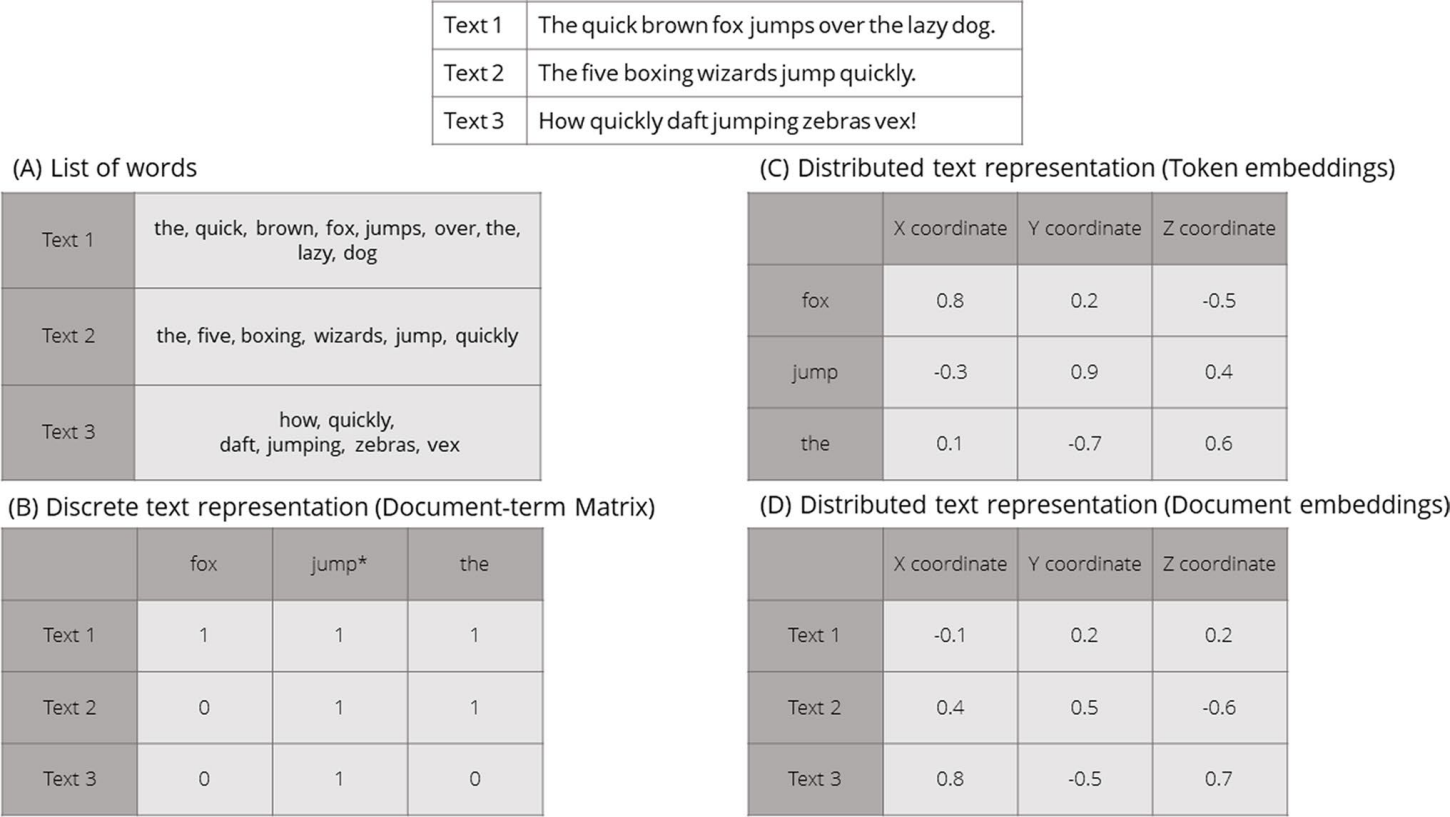
A visualization of different text representation methods

Before being fed into a text mining model, text needs to be pre-processed and converted into an appropriate input format (i.e., representation). In Fig. 1, we show the logic behind several approaches to text representation. Dictionary methods typically seek textual elements that match those contained in the dictionary (panel A in Fig. 1). These elements, often referred to as tokens, may comprise parts of a word, individual words, or multiple words. For the sake of simplicity, we will refer to tokens as words unless otherwise specified.

Machine learning models require words to be transformed into a numerical representation that captures word frequencies and/or relationships between words in each text. That is, a word needs to be represented as a point in a multidimensional semantic space so that its relationships with neighboring words are captured in a systematic manner (Jurafsky & Martin, 2021). The simplest approach relies on discrete “bag-of-words” representations, where each text is represented as a vector of frequencies of individual words it contains (panel B in Fig. 1). As a result, a textual corpus is converted into a document-term matrix (DTM), where rows represent each text (document), columns denote words occurring across texts (terms), and cells count word occurrence frequencies within texts (Aggarwal, 2018).^8^

Although valuable in many applications, discrete representations result in sparse vectors with many zero values (i.e., most words contained in a corpus appear in just a few texts) and disregard the context in which individual words appear (Jurafsky & Martin, 2021). This issue can be addressed by using distributed text representations. Such representations generate dense vectors that capture the context of each word (Mikolov, Sutskever et al., 2013b).

They can be obtained using word embedding models which represent words in a high-dimensional space, positioning words appearing in similar contexts close to each other in this vector space (panel C in Fig. 1, with a three-dimensional vector space as an example) (Mikolov, Chen et al., 2013a; Mikolov, Sutskever et al., 2013b; Vaswani et al., 2018).

In addition to such representations^9^, newer large language transformer models are also able to capture the fact that the same word can appear across different contexts (e.g., tree bark compared to a dog’s bark) (Vaswani et al., 2018). Some embedding models can further combine individual word representations to obtain representations for each sentence or text (panel D in Fig. 1).

In order to produce text representations, word embedding models are pre-trained on large textual datasets. The BERT model we evaluate was trained on the 800-million word BookCorpus and the 2.5-billion-word English Wikipedia corpus (Devlin et al., 2019); RoBERTa was trained on the same data, as well as the CC-News dataset containing 63 million English news articles, the OpenWebText dataset containing data from Reddit, and a subset of the CommonCrawl data (Liu et al., 2019); BERTweet was trained on a corpus of 850 million English Tweets (Nguyen et al., 2020). The data that GPT-3.5-turbo and GPT-4 models were trained on is not fully disclosed, but it likely involves more data from the internet in addition to the English Wikipedia, two internet-based book corpora, the CommonCrawl, and the WebText dataset (Brown et al., 2020).^10^

Here, we survey how text representations learned by these models can be used in conjunction with supervised classification (to help the model map relationships between vectors and coding categories based on manually coded data) or to help the model generate coding categories in the zero-shot classification approach.

Before being transformed into a representation, text can be pre-processed in order to drop redundant information. Usually, this includes lowercasing and removing punctuation. Next, one can choose to perform stop word removal—excluding short words that carry little meaning (e.g., “the”)—and text lemmatization*—*consolidating word forms and tenses into their root form.^11^ Past research has noted that pre-processing can significantly affect model performance, with optimal combination of steps depending on the particular application (Aggarwal, 2018; Denny & Spirling, 2018; Kern et al., 2016; Maier et al., 2018; Sun, 2012; Uysal & Gunal, 2014). Therefore, we experiment with multiple pre-processing steps where applicable (CMDs, logistic regression, RF, and SVM algorithms). When working with large transformer models for SML and zero-shot classification, we use the same pre-processing steps that were used during original model pre-training. In Table 1 we show combinations of methods and text representations we evaluate here. There are other possible combinations we do not survey (e.g., using dictionary output or distributed representations as input for simpler SML methods or enriching dictionaries using distributed representations).

**Table 1:** Tested representation and pre-processing combinations per method

Family	Method	(1) List of tokens	(2) Discrete representation (DTM)	(3) Distributed representation
**Dictionary methods**	RMD	List of tokens		
	CMD	(1) Lemmatization (2) Stop word removal		
**SML**	Logistic regression		(1) Lemmatization (2) Stop word removal	(see Appendix C)
	Random forest (RF)		(1) Lemmatization (2) Stop word removal	
	Support vector machines (SVM)		(1) Lemmatization (2) stop word removal	
	Multilabel random forest (Multilabel RF)		(1) Lemmatization (2) Stop word removal	
	Transformer models			(1) BERT (2) RoBERTa (3) BERTweet
**Zero-shot** **classification**				(1) GPT-3.5-turbo (2) GPT-4

### Step 4: Model parameter choices

We briefly discuss several challenging decisions related to model specification and present the choices we make in our systematic evaluation. Appendix C contains more details on the technicalities of our implementations.

#### Dictionary methods

Researchers might be able to choose among several available RMDs in some applications, whereas others might call for theoretically informed manual selection of words of interest for each category. When creating CMDs using our procedure, the first step entails establishing the cutoff values for the keywords that will be included in the dictionary. As we work with relatively small datasets, we set the *p*-value cutoff for the log-likelihood measure to 0.01, which is higher than the cutoffs in work handling corpora with millions of words (Pojanapunya & Watson Todd, 2018). When working with effect size measures, we set the cutoff to words in the top 20% of the distribution of the %DIFF scores. Once the CMD is generated, researchers need to select the classification threshold, specifying how many words from a dictionary category must be present in a text for it to be classified into the corresponding category. As we are working with short texts, we set this threshold to at least one word from a category. However, optimal parameter choices might differ depending on the specific application (also see Appendix F, where we vary word cutoffs and coding thresholds).

#### SML classification methods

SML methods include numerous parameters which, if adjusted to the task at hand, could affect model performance. As these parameters are specific to each model, we do not discuss them in detail here and mostly rely on the default settings in the software implementations we use. Researchers can use automatic methods for hyperparameter tuning to seek the best parameter combination for each model and application (Feurer & Hutter, 2019). In our logistic regression analyses, we use ridge regression to prevent model overfitting due to the large number of textual features (Craig et al., 1998). Logistic regression outputs whether the text belongs to a category or not (0/1). When working with RF and SVM, we request models to output the probability of each text belonging to each category and classify texts into a category if this probability is above 0.5.^12^

When fine-tuning autoencoding transformer models (BERT, RoBERTa, and BERTweet) using our manually coded data, we modify both the model parameters and the classification part of the model (i.e., classification head) (C. Sun et al., 2019).^13^ We fine-tune each model for five epochs.^14^ Then we transform the outputs of the classification head (logits) into probabilities (see Appendix B for details), applying the same classification logic as for the other SML methods.

#### Zero-shot learning

When evaluating GPT-3.5-turbo and GPT-4, we use the OpenAI (the company developing these models) API to feed instructions to the model. Various strategies for designing these model instructions (i.e., prompts) exist (Liu et al., 2021; Wang et al., 2023). While there is evidence that different prompting strategies affect model performance (Abdurahman et al., 2023; Reiss, 2023), we chose to keep our prompts minimal. We provide the model with the text source and coding categories only and give it an instruction that it should be acting as “...a research assistant” (see Appendix B for details). We do not change other default parameters.

### Step 5: Performance evaluation

**Fig. 2:**
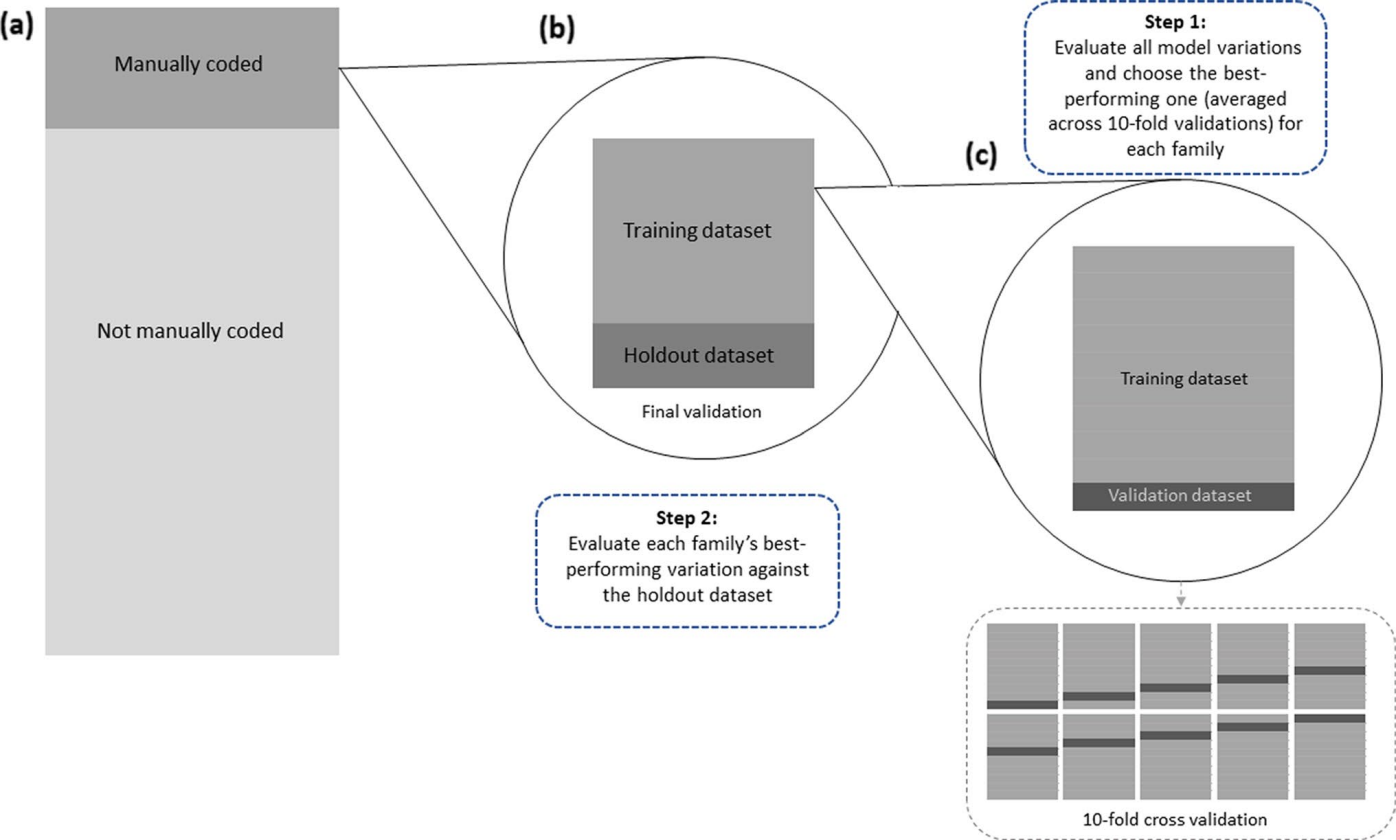
A schematic representation of our performance evaluation procedure. We first manually code a sample of texts (a) and then designate 80% of the sample as the "training dataset" and the remaining 20% as the "holdout dataset" (b). We implement a 10-fold cross-validation approach (c) to choose the best-performing model variant within each method family before evaluating each family's performance on the "holdout dataset".

The performance of text mining methods that use manually coded data as input should not be evaluated against the same data used when the model was built (or fine-tuned). Figure 2 summarizes our evaluation procedure: After selecting the sample of texts and coding it manually (Fig. 2a), we designate a randomly selected portion of this sample (80%) as the “training” set that will be used to build or fine-tune our models. We reserve the remaining 20% of the sample as a “holdout” set for evaluating model performance against the gold standard of human coding (Fig. 2b) (Raschka, 2020). Since the performance of models trained on manually coded data can vary depending on the random selection of texts into the training set (James et al., 2013), we additionally implement a 10-fold cross-validation approach (Fig. 2c). The training data are randomly shuffled and split into 10 subsets, and each subset is then used to evaluate the model trained on the remaining nine subsets (James et al., 2013).

We average the performance of each model variation across 10 validations to determine the best-performing variation within each family (CMD and SML methods). We then train this variation on the complete training dataset and evaluate its performance on the holdout dataset. For models that are not trained on manually coded data (RMD and zero-shot classification), we only evaluate their performance on the holdout set, comparing it to the manually coded gold standard. The method that performs the best on the holdout set can then be used to automatically code new data (Fig. 2a, data that have not been manually coded).

The simplest measure of performance against the gold standard is accuracy. Accuracy measures the proportion of texts for which the coding done by the model matches human coding (Aggarwal, 2018). In some cases where texts can simultaneously belong to multiple categories, we also report subset accuracy, which denotes the share of texts for which all categories were predicted correctly (Nam et al., 2017). However, relying on accuracy only can mask poor performance on rarely occurring coding categories.^15^ Therefore, we also report the F-score which captures success in identifying the presence of coding categories. The F-score is a harmonic mean of precision (the percentage of texts the model identified as belonging to a category that actually belong to it as per the gold standard) and recall (the percentage of texts belonging to the category as per the gold standard that the model identified as such) (Aggarwal, 2018). A higher F-score indicates better predictions, with 1 indicating a perfect match to human coding. To evaluate the performance across multiple categories in our coding schemes, we report micro-averaged F-scores.^16^

## Case studies

We use four datasets and coding schemes as case studies for our method comparison. These include a dataset of 2,000 texts from an online marketplace coded for seven motives of their authors, two Twitter datasets containing 3,832 and 24,783 texts and coded for moral norm expression and the presence of hate speech, respectively, and a dataset of 10,000 Reddit posts coded for the expression of six different emotions. We selected these datasets so as to cover a range of online platforms, average text lengths, sizes of manually coded datasets, and types of internal states in text. Table 2 lists dataset details, and Table 3 presents information about coding categories and the ranges of intercoder agreement on each of them. In Appendix B, we provide details on the individual coding schemes and procedures.

**Table 2:** Dataset information

	Feedback data	Election data	Reddit data	Hate speech data
**Texts**	2,000	3,832^a^	10,000^b^	24,783^c^
**Avg. words**	13.3 (*SD* = 11.9)	14.8 (*SD* = 6.7)	13.3 (*SD* = 6.8)	13.6 (*SD* = 7.0)
**Coded for**	Motives	Moral norms	Emotions	Hate speech
**Categories**	7	10	6	3
**Classification problem**	Multi-label^d^	Multi-label^d^	Multi-label^d^	Multi-class^d^
**Data source**	(Norbutas et al., 2020)	(Hoover et al., 2020)	(Demszky et al., 2020)	(Davidson et al., 2017)
**Platform**	Online market	Twitter	Reddit	Twitter

^a^ The original election dataset coded by Hoover et al. (2020) contained 5,358 tweets. However, we were unable to retrieve all the original tweets via Twitter API; some of the tweets or accounts tweeting them appeared to be deleted at the time of our retrieval (third quarter of 2021).

^b^ The original dataset contains 58,011 coded tweets; we selected a random subsample for computational feasibility. We chose a sample of 10,000 texts as an intermediate size between the smaller Feedback and Twitter datasets and the larger Hate speech dataset.

^c^ The original paper mentions 24,802 texts; we obtained 24,783 tweets from the publicly available dataset.

^d^ Multi-label implies that a single text can contain multiple categories at once; multi-class suggests a text can belong to only one of multiple categories.

**Table 3:** Coding categories, category prevalence, and coder agreement per category

Dataset	Coding categories	% of texts coded for (min–max)^a^	Fleiss’ kappa (min–max)^b^	PABAK (min–max)^b^
Feedback data	Reach out to the seller, Share objective facts, Express feelings, Help the seller, Avoid harming the seller, Help other buyers, Reward or punish the seller	3.70–69.80	.25–.53	.42–.90
Election data	Care, Harm, Fairness, Cheating, Loyalty, Betrayal, Authority, Subversion, Purity, Degradation, Non-moral	2.11–56.24	.18–.45	.73–91
Reddit data	Anger, Disgust, Enjoyment, Fear, Sadness, Surprise	1.34–40.08	.27–.53	.54–.95
Hate speech data	Hate speech, Offensive language, Neither	5.77–77.43	.55	.72

^a^ The % of texts coded for the least and most frequently occurring coding categories, respectively

^b^ The lowest and highest values, respectively, of Fleiss’ kappa/PABAK across the categories in the coding scheme

In the first application with our own data, we investigate motivations to write feedback after engaging in a transaction in an online marketplace for illegal goods. Feedback plays an essential role in the functioning of large-scale online marketplaces. In this dataset, we seek cues left by feedback text authors as to why they chose to invest their time and effort to write a detailed report on their experience. We sample 2,000 feedback texts and have each text coded independently by three trained coders. Our coding scheme includes seven coding categories capturing different motives for writing (Macanovic & Przepiorka, 2023). We refer to this dataset as the “Feedback data”.

In the second application, we use a set of tweets related to the 2016 presidential election in the United States collected by Hoover and colleagues (2020). This set is a segment of a larger corpus of tweets manually coded for the expression of moral sentiments of care, fairness, loyalty, authority, and purity as per Moral Foundations Theory (Garten et al., 2016; Graham et al., 2013). These data offer valuable insights into the moral backdrop of public discussions during the tumultuous 2016 presidential elections. We refer to them as the “Election data”.

In the third application, we select a random subset of 10,000 forum posts (from the original dataset of 58,011 posts) from Reddit, a large social platform. These Reddit posts were manually coded for 27 different emotions by Demszky and colleagues (2020). For simplicity, we compile these emotions into six general categories as per the Ekman universal emotions categorization (Ekman, 1992). We refer to this dataset as the “Reddit data”.

Our final application includes a set of 24,783 tweets coded for the presence of hate speech, offensive language, or neither of the two by Davidson and colleagues (2017). Unlike the previous three datasets, where each text could contain multiple categories at once (multi-label classification), in this scheme, each text could only be coded for one of the three categories (multi-class classification). We refer to this dataset as the “Hate speech data”.

## Results

### Overall performance

Table 4 shows the performance of the best-performing variation in each method family against the human-coded reference on the holdout dataset. In Appendix D, we report alternative performance measures. In Appendix E, we report extensive results on the performance of different model variations. We discuss micro-averaged *F*-scores unless otherwise noted. Table 4 shows that fine-tuned transformer models outperform RMDs, CMDs, and zero-shot classification. CMDs take second place in all applications but the Hate speech one, where they are outperformed by zero-shot classification. Depending on the dataset, RMDs can perform better or worse than some CMD variants, but they are always outperformed by the best-performing CMD.

**Table 4:** Best-performing methods within each text mining method family

	**Feedback data**				**Election data**			
**Family**	**Method**	**F-score**	**Acc.**	**Subset acc.**	**Method**	**F-score**	**Acc.**	**Subset acc.**
RMD	Dict. 1	.329	.800	.152	Dict. 1	.492	.875	.312
CMD	Dict. 2	.537	.806	.260	Dict. 3	.477	.879	.298
SML	RoBERTa	.779	.919	.562	RoBERTa	.696	.939	.550
Zero-shot	GPT-4	.499	.666	.032	GPT-4	.409	.836	.227
	**Reddit data**				**Hate speech data**^**a**^			
**Family**	**Method**	**F-score**	**Acc.**	**Subset acc.**	**Method**	**F-score**	**Acc.**	**Subset acc.**
RMD	Dict. 1	.369	.734	.195	Dict. 1	.611	.611	*NA*
CMD	Dict. 3	.501	.837	.376	Dict. 3	.845	.845	*NA*
SML	RoBERTa	.690	.927	.626	RoBERTa	.907	.907	*NA*
Zero-shot	GPT-3.5	.492	.861	.386	GPT-4	.897	.897	*NA*

All performances are reported on the holdout dataset, which is identical for each method

^a^ In the Hate speech dataset, different categories cannot co-occur in a single text, which is why micro-averaged score and accuracy take the same values and the subset accuracy is not calculated

While the zero-shot approach relying on the more recent GPT-4 model outperforms RMDs and its predecessor (GPT-3.5-turbo) in most cases, its performance falls short of transformer models fine-tuned with manually coded data. Notably, GPT-4 performs worse than an extensively validated RMD on the Election data. While the performance of zero-shot classification can potentially be improved by adjusting the prompt or providing several examples (few-shot learning), this requires additional considerations regarding prompt design and example selection (Abdurahman et al., 2023; Chae & Davidson, 2023; Liu et al., 2021). These findings underscore some of the constraints of using readily available RMD resources and zero-shot classification methods when assessing certain internal states within short, informal texts from online platforms.

The subset accuracy scores in Table 4 indicate the percentage of texts for which all categories were correctly predicted, indicating how successful each method is in capturing category co-occurrence. Fine-tuned transformer models fare much better than all other methods when it comes to accurately predicting co-occurrences within texts.

**Fig. 3:**
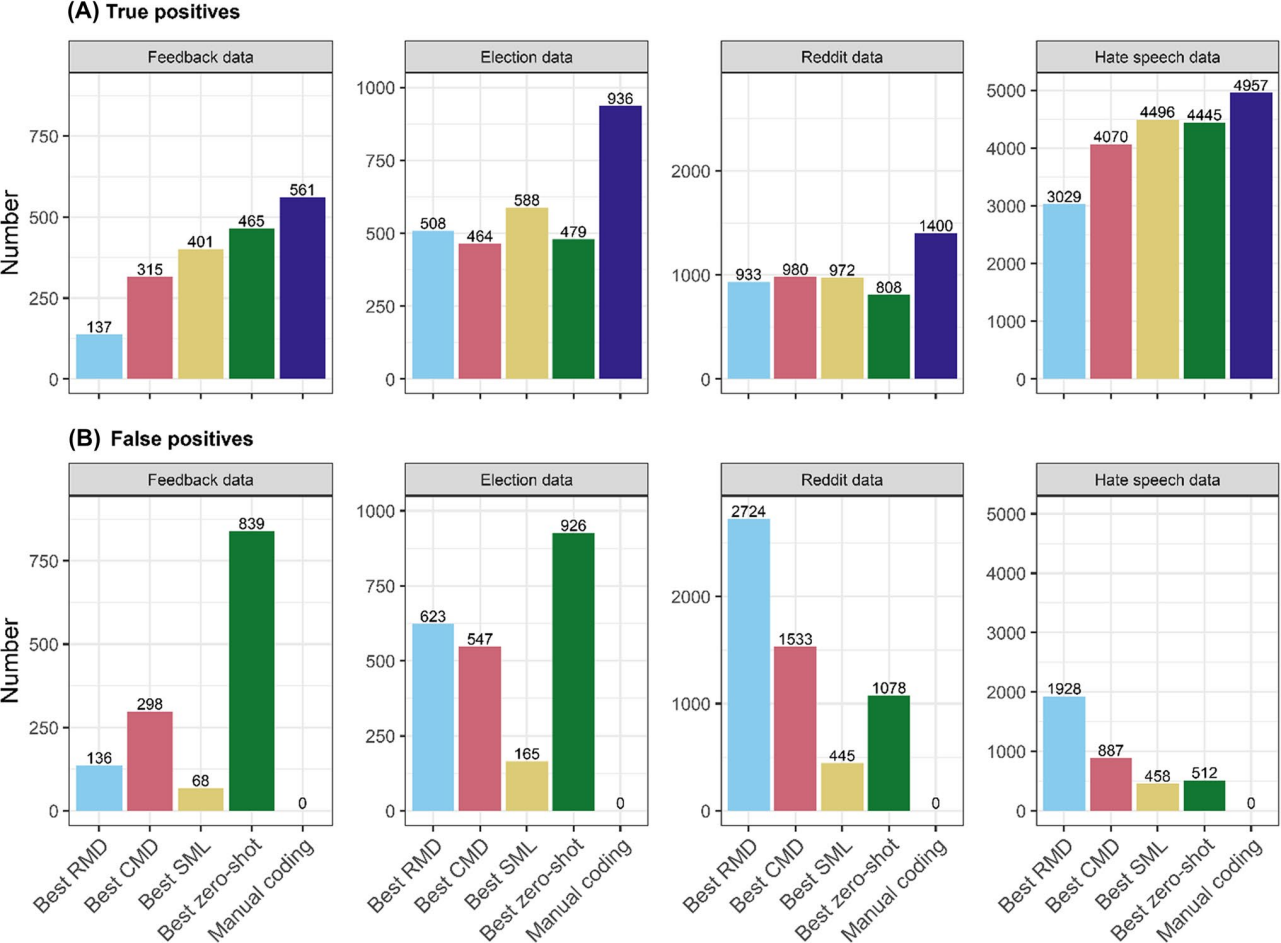
Comparison of model performance in identifying true positives and raising false positives

To provide an intuitive interpretation of the F-scores above, in Fig. 3 we show how the best-performing model from each family fares in terms of identifying true positives (matching manual coding for a category) and avoiding false positives (identifying texts not manually coded for that category). Taking Feedback data as an example, human coders indicated the presence of 561 coding categories across 400 texts in the holdout dataset (with some texts containing multiple categories at once). The best-performing SML model (RoBERTa) correctly identified 401 of these texts (true positives), but incorrectly flagged 68 texts for categories they were not coded for by the human coders (false positives). Overall, we see that, while they can perform well in terms of identifying true positives (Election and Reddit data), RMDs flag many more false positives than CMDs and SMLs (except in Feedback data). Notably, the best zero-shot classification model similarly returns almost twice as many false as true positives on some datasets (e.g., Feedback and Election data). It is this proneness to false positives that lowers the performance of zero-shot approaches relative to fine-tuned transformers.

### Step 2: Method choice

#### Dictionary methods

**Fig. 4:**
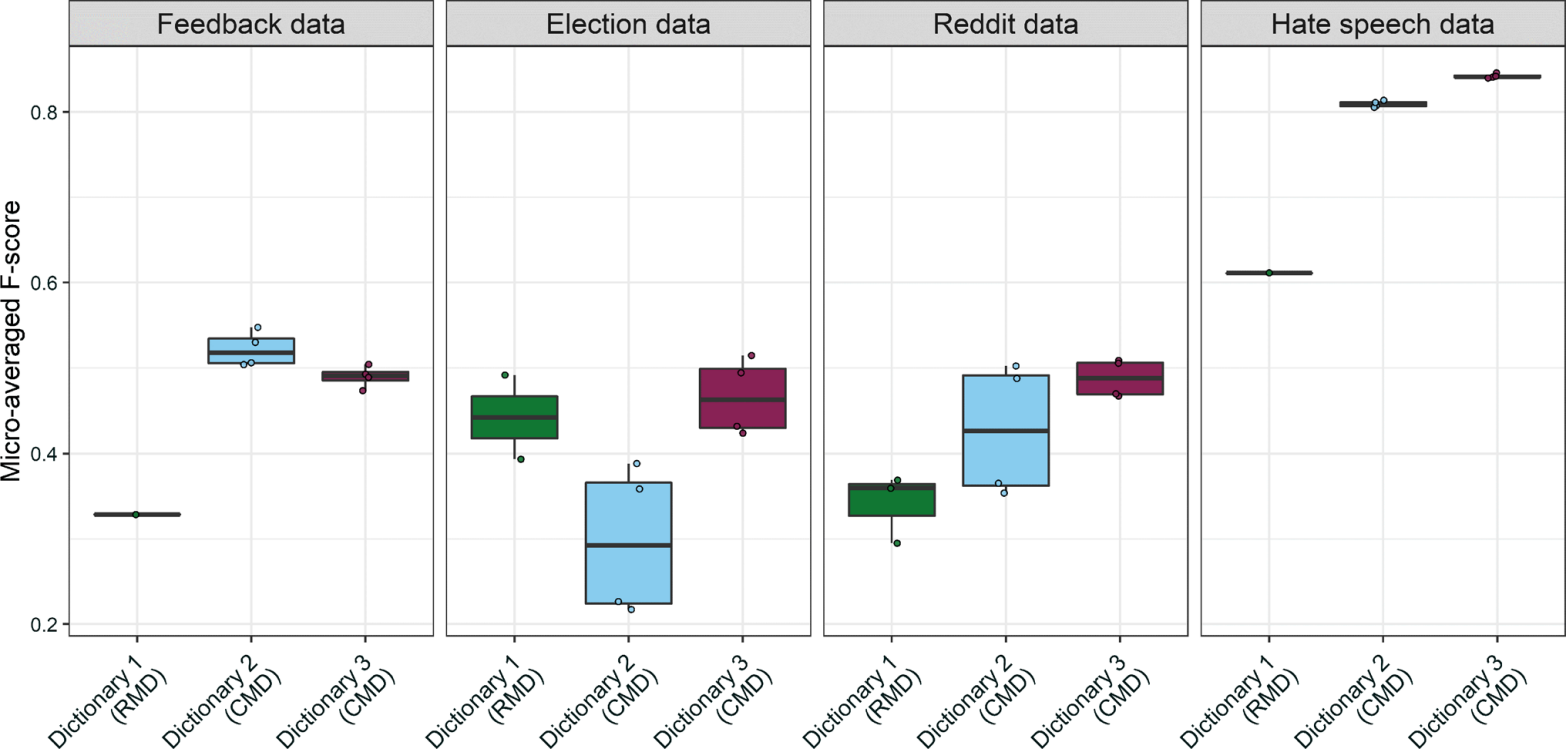
Performance of different dictionary method variations across four datasets

In Fig. 4, we show the performance of different dictionary method variations. We report the performance on the holdout set for RMDs. To illustrate the performance variation of different CMD variants, we report the averages across the 10 cross-validation rounds. In Appendix F we discuss the overlaps between words in different dictionaries.

On Feedback and Hate speech datasets, we evaluated only one RMD, as we failed to find more resources fitting these coding schemes. For the former, we create a dictionary from several existing resources (including the Moral Foundations Dictionary 2.0, the LIWC, and the words we find relevant based on our framework and experience with the data, see Appendix B). For the latter, we use the HurtLex dictionary of offensive, aggressive, and hateful words in English, using the insults and misogyny categories for offensive language and immigrant and xenophobia categories for hate speech (Bassignana et al., 2018). On the Election dataset, we evaluate the Moral Foundations Dictionary 2.0. (Frimer et al., 2017) and the enhanced Moral Foundations Dictionary (Rezapour et al., 2019). Both dictionaries performed rather well, with the former approaching the best-performing CMD variations, and both RMDs outperforming the worst-performing CMD.

On Reddit data, we compare three RMDs that can identify emotions of interest as per the coding scheme: the NRC emotion dictionary (Mohammad & Turney, 2013), the WordNet-Affect dictionary (Strapparava & Valitutti, 2004), and the EmoSenticNet dictionary (Poria et al., 2013). The WordNet-Affect and EmoSenticNet dictionaries performed similarly to some of the CMDs with a lower performance. EmoSenticNet performed the best on this dataset, while the NRC lexicon performed the worst. Overall, CMDs delivered better performance than RMDs. There is no clear case of either the statistical significance (Dictionary 2: log-likelihood) or the effect size measure (Dictionary 3: %DIFF) performing better overall. Rather, we find that the performance varies with the dataset and the pre-processing combination. Additionally tuning word selection or classification thresholds could lead to further improvements in CMD performance.

What distinguishes RMDs that performed well from those that did not? We find that RMDs perform rather well if they were created with a similar (or identical) coding scheme in mind and extensively validated, as was the case with the dictionaries created to capture Moral Foundation Theory categories across many domains. For instance, the Moral Foundations Dictionary 2.0 included many words that we selected for our CMDs. On the Reddit dataset, the worst-performing NRC lexicon created from the Macquarie Thesaurus (Mohammad & Turney, 2013) included many words related to general emotion expression, but lacked those characteristic of the informal online context. On the other hand, EmoSenticNet and WordNet-Affect were both built on a large lexical database of English (Miller, 1998) and, thus, included more words that appear in our dataset. While NRC includes words such as “whimsical” or “pleased” in relation to “joy,” the other two RMDs also feature “cool” and “awesome.” Additionally, our CMD included the internet-specific “lol,” which is likely to be particularly relevant when analyzing Reddit posts.

#### SML methods

**Fig. 5:**
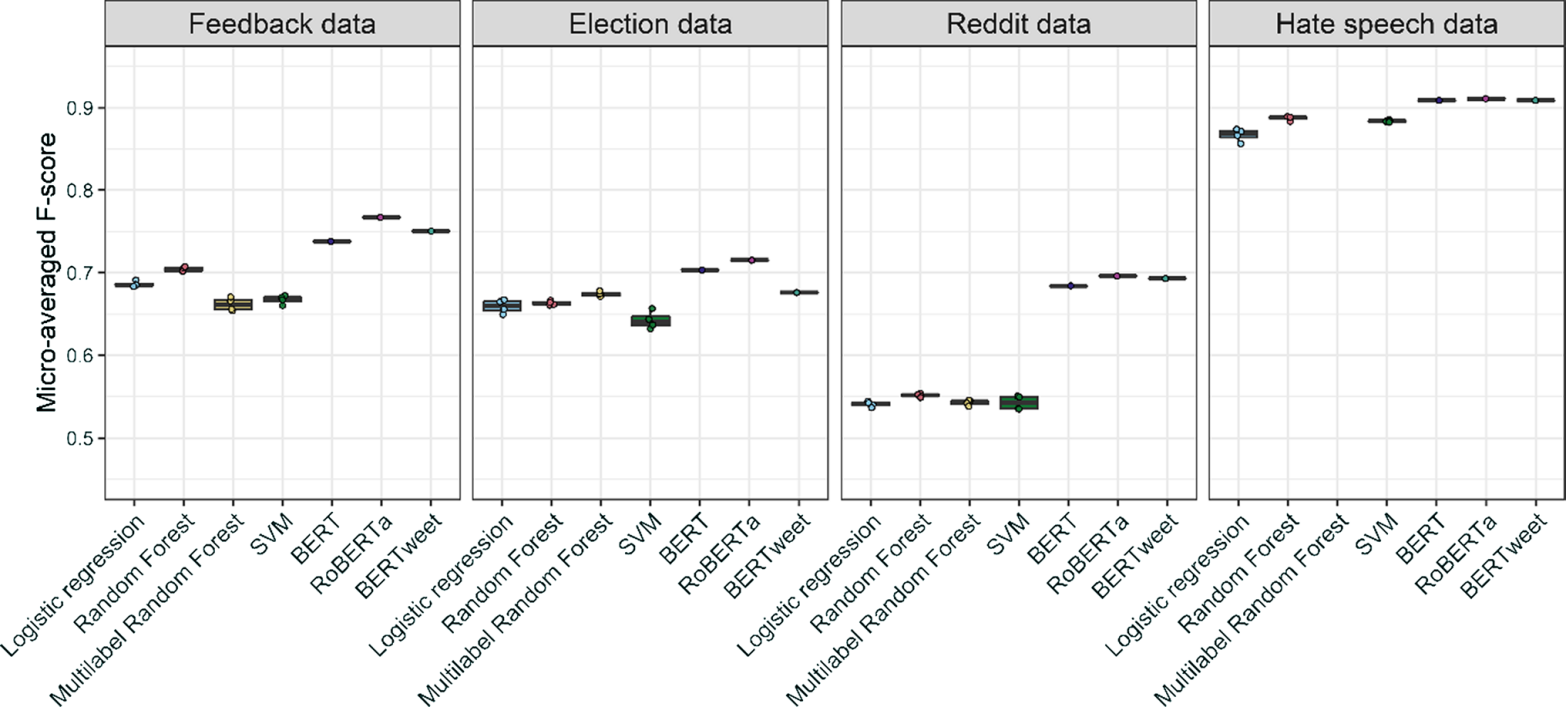
Performance of different SML method variations across four datasets

In Fig. 5, we show the performance of various SML model variations averaged across 10 cross-validations. Fine-tuned transformer models consistently outperform simpler SML algorithms. Among the transformer models, RoBERTa outperforms BERT and BERTweet. However, the differences in performance between transformer models tend to be small (especially on the Reddit and Hate Speech datasets with larger training datasets).^17^

**Fig. 6:**
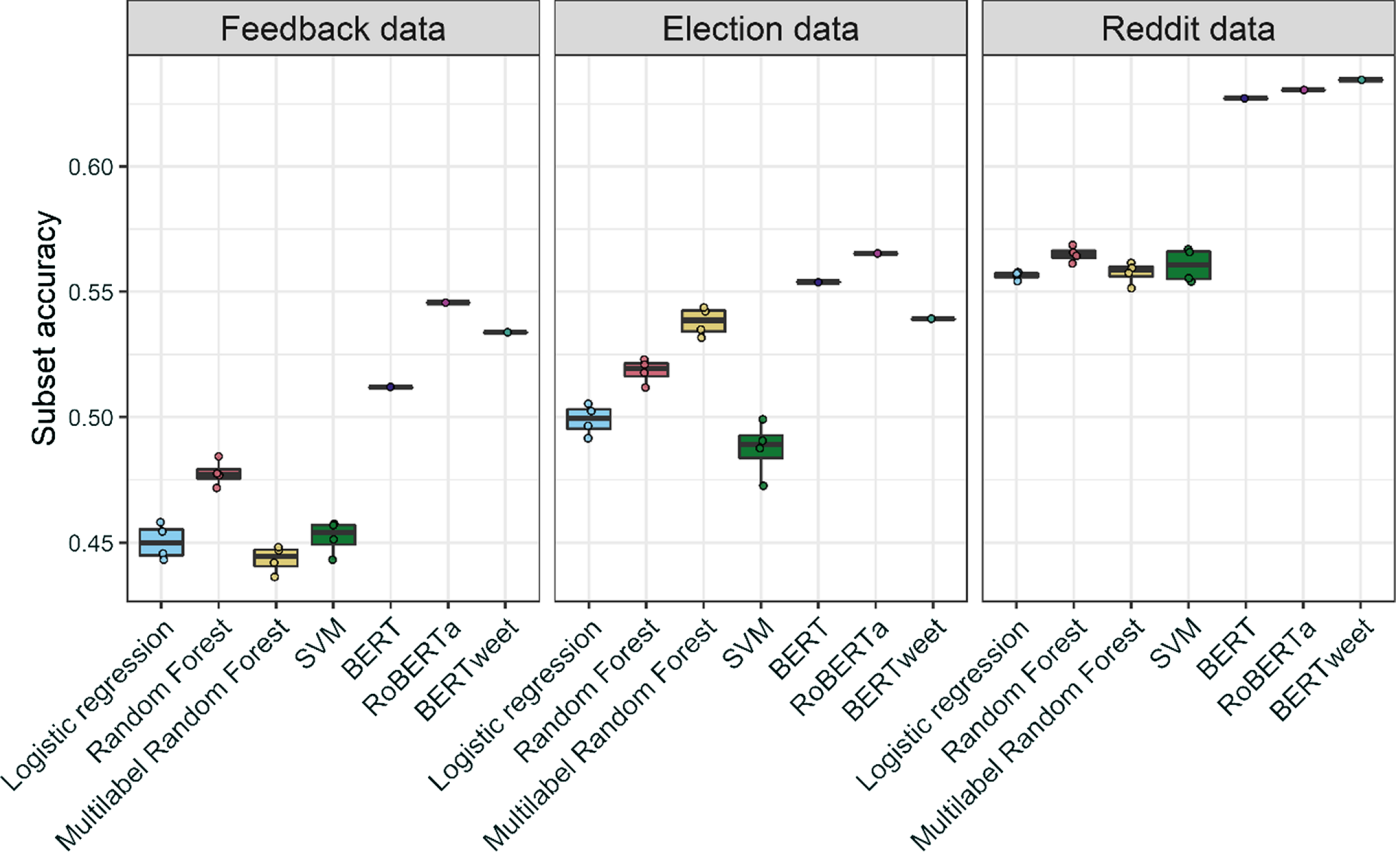
Performance of different SML method variations across four datasets: Subset accuracy

Simpler SML models lag behind fine-tuned transformers in all datasets, although the gap is rather small in the Hate Speech dataset. Among them, logistic regression and RF implementations perform the best. On the Election and the Hate speech data in particular, simpler SML algorithms approach the performance of the more complex transformer models. Yet, Fig. 6 plotting subset accuracy (the share of texts for which all coding categories were predicted correctly) shows that logistic regression performs rather poorly in identifying category co-occurrences. The multi-label RF algorithm, designed with co-occurrences in mind, outperforms RF only on the Election dataset. In Figure E4 in Appendix E, we see that the Election data show a more complex category co-occurrence pattern than the other two datasets. Therefore, in applications where capturing co-occurrences correctly (e.g., knowing that a text expresses both joy and surprise) is crucial to the research question, algorithms designed to model category interdependence should be considered as well.

#### Zero-shot classification

**Fig. 7:**
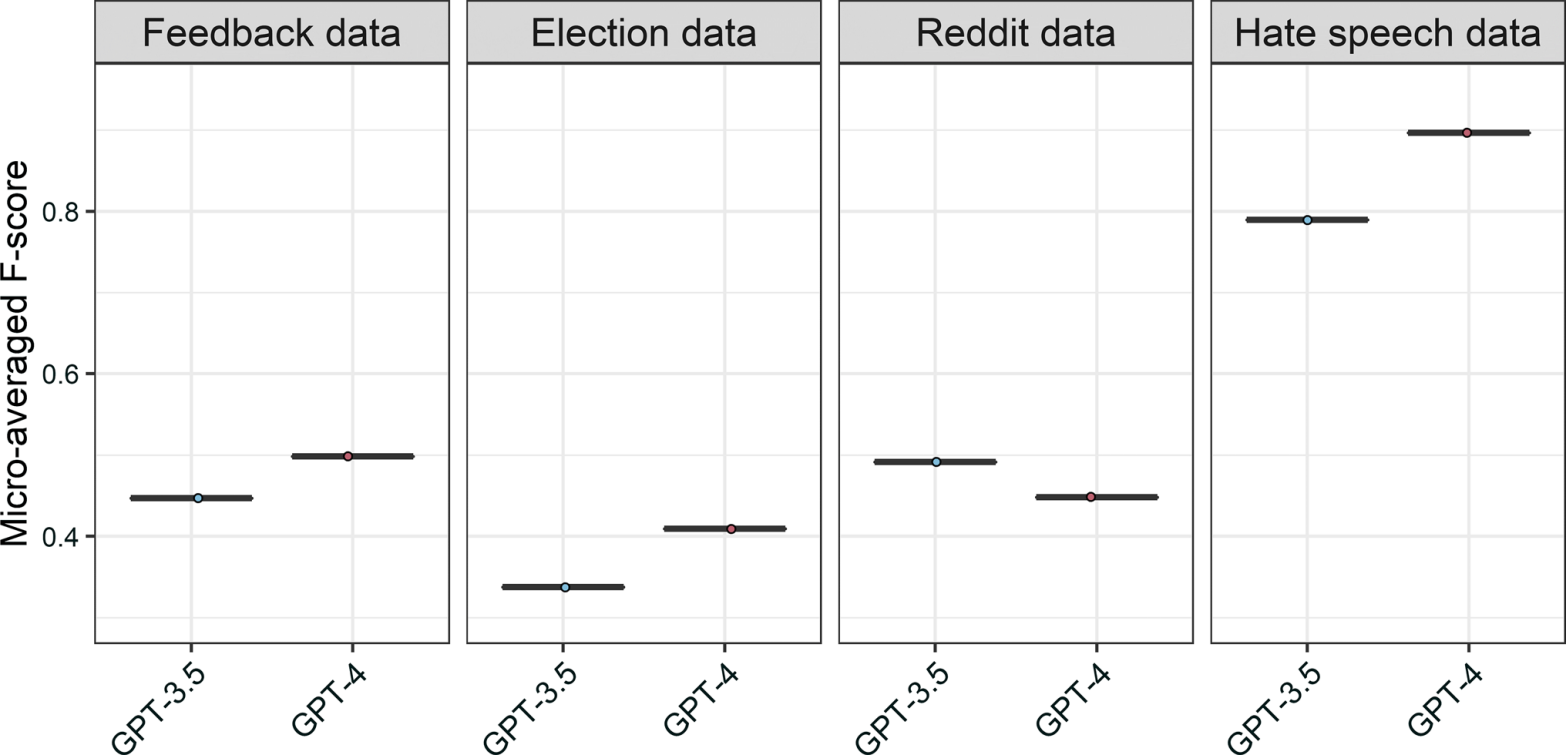
Performance of different zero-shot classification models across four datasets

In Fig. 7, we show the performance of zero-shot classification. The newer GPT-4 model performed better than its predecessor, the GPT-3.5-turbo, on all datasets except the Reddit dataset. On the Hate speech dataset, GPT-4 in particular performs on par with fine-tuned transformer models, suggesting that zero-shot classification can handle some tasks rather well. Providing GPT-4 with more thorough instructions akin to those in extensive coding schemes could potentially lead to further improvement in its performance (see Figure E9 in Appendix E).

### Step 3: Data pre-processing

#### Dictionary methods

**Fig. 8:**
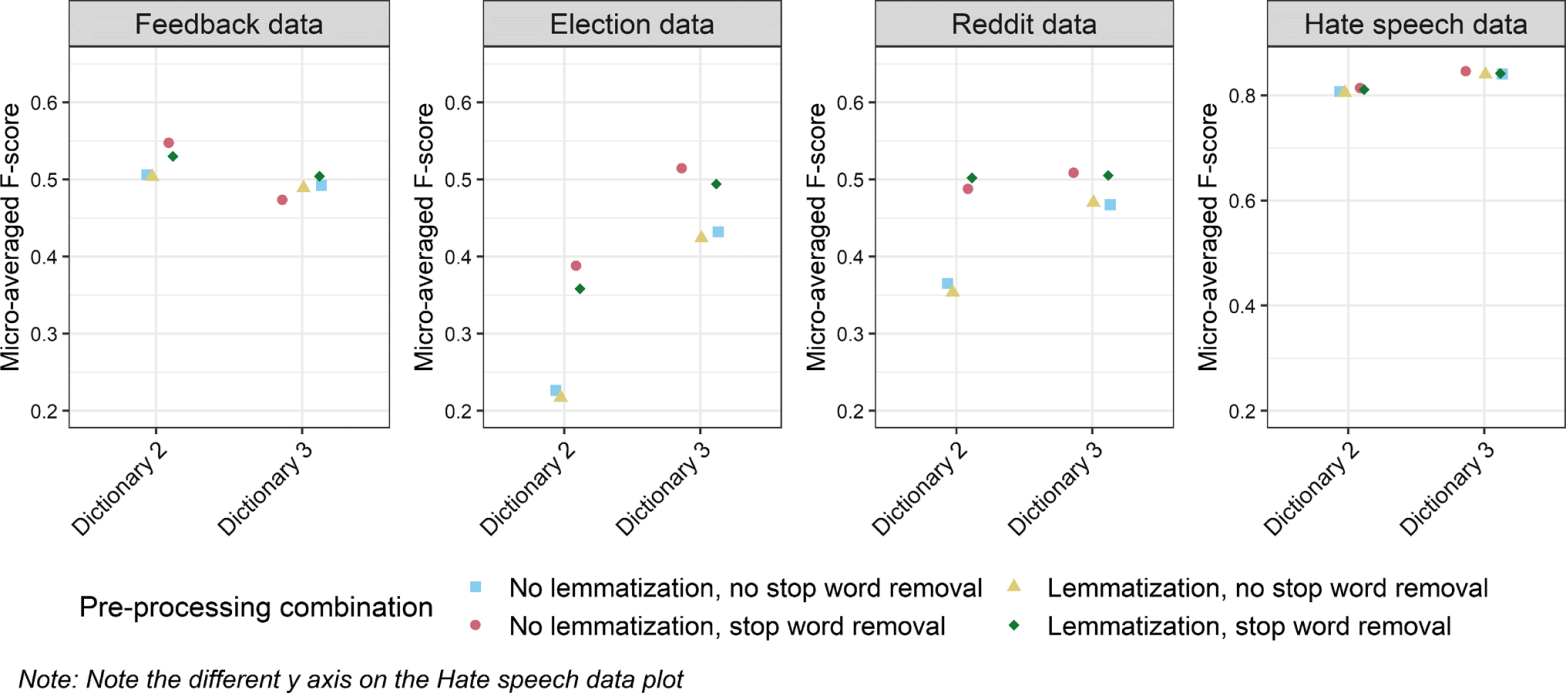
Performance of CMDs built on differently pre-processed text

In Fig. 8, we show the performance of CMDs built on differently pre-processed data. On the whole, CMDs built on data with stop words removed tend to perform better, whereas the effect of lemmatization depends on the dataset. Overall, the performance of Dictionary 3 (%DIFF measure) appears more robust to variations in pre-processing combinations.^18^

#### SML methods

**Fig. 9:**
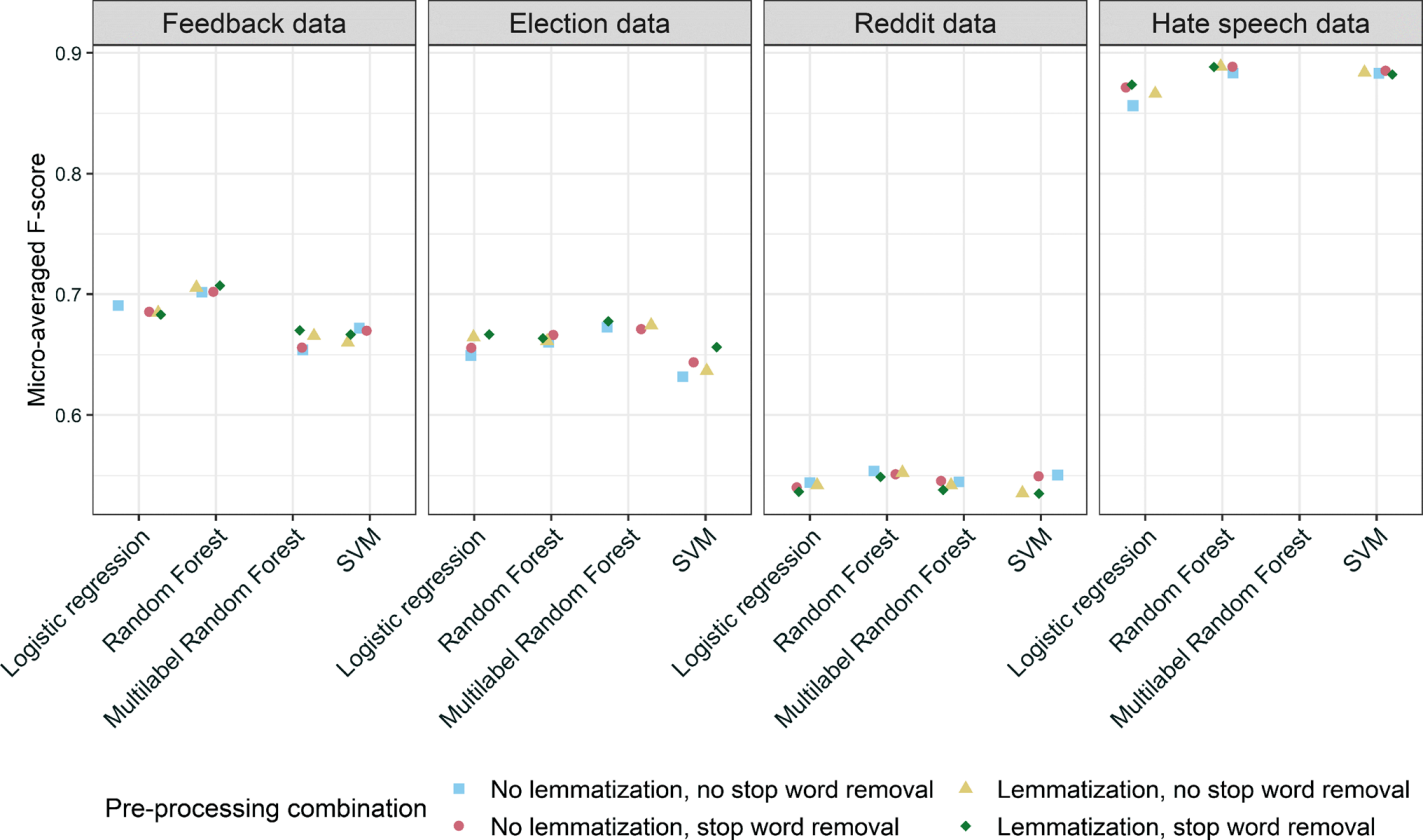
Performance of SML models built on differently pre-processed text

In Fig. 9, we show the effects of data pre-processing on SML performance (excluding the transformer models with dedicated preparation pipelines). The performance of SML classification methods appears robust to different pre-processing combinations. While it does appear that lemmatization improves model performance in most cases, performance differences remain relatively small. In Appendix E, we discuss several additional pre-processing steps and alternative text representations and evaluate how they affect model performance.

### Performance per coding category

**Fig. 10:**
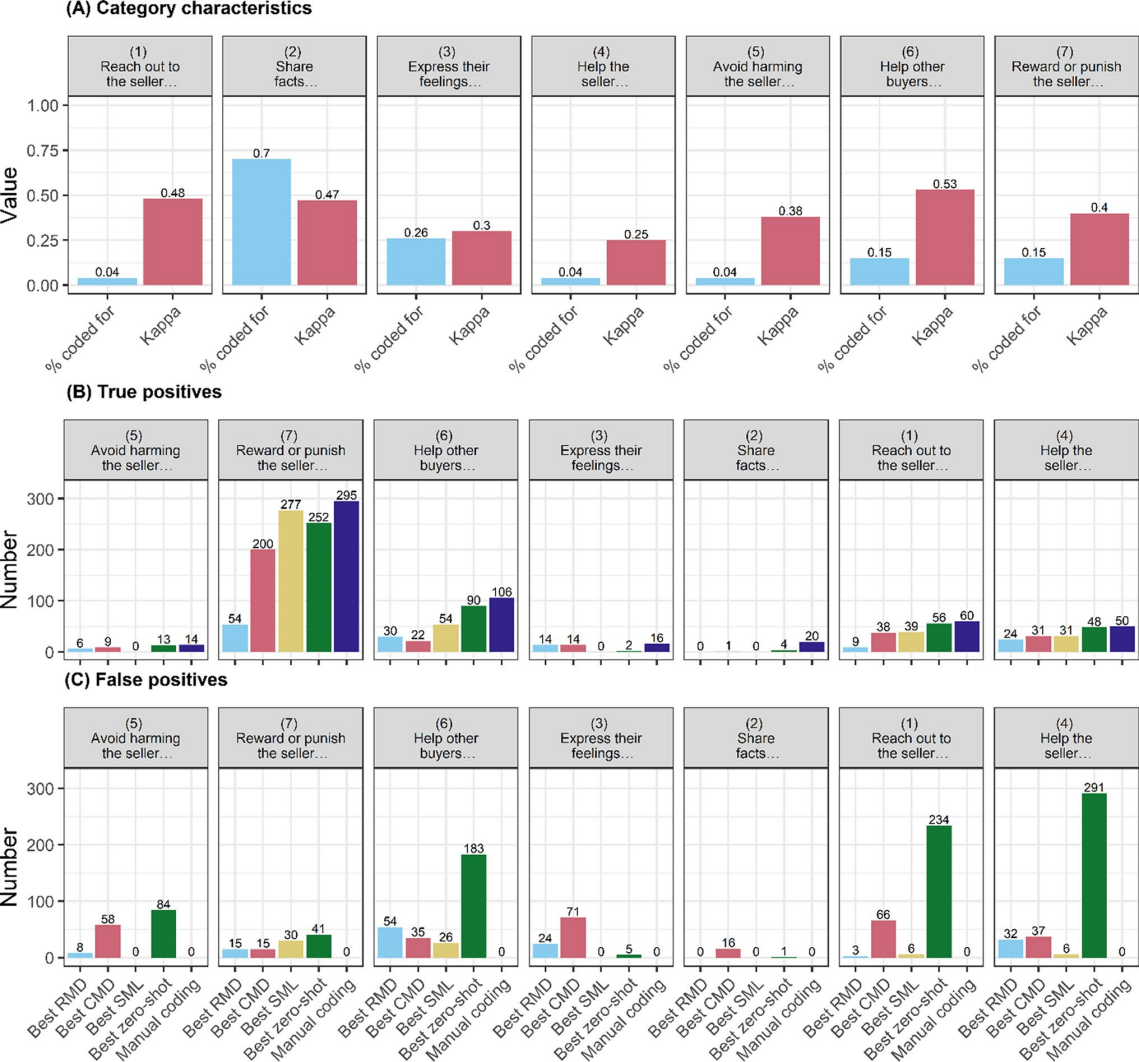
Performance per coding category: Feedback data

We now briefly reflect on the performance of different methods in coding for individual categories. In Fig. 10, we show the performance of the best-performing method from each family in identifying true positives (panel B) and avoiding false positives (panel C) in each of the coding categories on the Feedback data (in Appendix G, we do the same for the Election and Reddit datasets). In panel A, we show the prevalence of each category (% of texts coded for its presence) and the value of Fleiss’ kappa (intercoder agreement). We see that the best-performing transformer SML model fails to identify infrequently occurring categories altogether (i.e., categories 1, 4, and 5). The literature advises researchers to lower the classification probability threshold for infrequently occurring categories (Zou et al., 2016). However, we did not see an improvement upon doing so with the RoBERTa model on Feedback data.

Intercoder agreement seems to correlate with the success of the SML transformer models in identifying categories to a lesser extent: for instance, while categories 3 and 4 have similar levels of agreement, the more frequent category 3 presents less of a challenge for the SML algorithm (but see Wang et al., 2022). In particular, we see that our best-performing CMD identifies true positives on these rare categories much better than the SML method, but does so at the risk of identifying (many) false positives. The best zero-shot model similarly identifies some infrequent categories better than the SML, but can also flag many false positives. If identifying infrequent categories is important, complementing SML models with CMDs might prove fruitful. Zero-shot models might also be useful for exploring infrequent categories, but as they are rather nontransparent in their decisions, CMDs might be a more tractable choice in many cases.

## Conclusions and final remarks

The ability to analyze large quantities of textual data becomes increasingly relevant in a world where individuals generate millions of texts every day. These texts, written in natural settings, are an excellent source for inference on individual-level processes. We hope our survey will help social and behavioral scientists navigate the rapidly changing landscape of computational text analysis methods and identify best practices for their particular case studies. We provide concise advice on selecting methods that can reliably extend manual coding for different internal states onto large amounts of text, and highlight those cases where commonly used resources (such as the pre-existing dictionaries) and new, increasingly popular approaches (such as zero-shot classification with GPT models) might do well or fall short of our expectations and needs. Below, we summarize the main conclusions of our survey and outline the trade-offs that should be considered by researchers who are working with these methods.

### Which method family compares to human coding the best?

We find that fine-tuned transformer models achieve the highest performance compared to the gold standard of human coding on all of our tasks. These findings confirm recent suggestions on their potential in social science applications (Bonikowski et al., 2022; Do et al., 2022; Torres & Cantú, 2022; van Atteveldt et al., 2021; Widmann & Wich, 2022). While transformers outperform simpler supervised algorithms and bag-of-words representations, some simple models such as logistic regression can perform quite well, particularly on tasks where coding categories rarely co-occur across texts. Finally, although zero-shot classification methods we tested were very easy to use, they did not perform as well as fine-tuned transformers. While they were successful in correctly identifying many coding categories, they also tended to be very sensitive and flagged many categories that should not have been flagged according to human coders. Dictionary methods tended to perform well on some (infrequent) categories, but also produced many false positives, even if tailored to a specific dataset and coding task (as was the case with the CMDs we tested). 

### Risks and benefits of using dictionary methods

We show that ready-made dictionaries can perform relatively well. This is especially the case for extensively validated domain-specific dictionaries that match the coding categories well. At the same time, our results highlight the need for caution when using ready-made dictionaries from a different domain to code short, informal texts for some internal states (Jaidka et al., 2020). Custom-made dictionaries derived from manually coded data tend to outperform the ready-made ones. The use of custom-made dictionaries provides researchers with transparency regarding coding decisions and proves particularly useful when identifying rare categories in text. However, like ready-made dictionaries, they lack the sensitivity of more advanced methods and, as a result, return many false positives. It is, therefore, crucial to validate dictionary analyses either through manual checks or, as we did, against a systematic manual coding reference (Grimmer et al., 2022; Grimmer & Stewart, 2013). Beyond this, dictionaries can also be used to seek new texts of interest in unstructured textual data (Eads et al., 2021; King et al., 2017).

### Risks and benefits of using zero-shot classification

Recent work has suggested that autoregressive generative large language transformer models (such as GPT) outperform some humans in coding quality (Gilardi et al., 2023; Törnberg, 2023) and show great potential for the classification of internal states in text (Rathje et al., 2023). Implementing zero-shot classification is much less technically demanding than transformer fine-tuning. Chat wrappers around some proprietary models (e.g., GPT-3.5-turbo and GPT-4) provide an easy way to communicate with the model in natural language, making the process even easier. Unlike open-source transformer models (such as BERT or RoBERTa), the use of these proprietary models is not free. However, if proprietary models excel in zero-shot classification, they have the potential to reduce the need for manual coding of hundreds or thousands of texts, thereby lowering the costs of reliable text analysis in the long term. We find, however, that zero-shot classification delivered lower performance and a stronger tendency for flagging false positives than fine-tuned autoencoding transformer models like RoBERTa. Recent studies suggest that adjusting prompting strategies or providing several high-quality examples can improve the classification performance of generative models (Abdurahman et al., 2023). Similarly, recent work suggests that fine-tuning the new generation of autoregressive generative models (such as GPT) calls for less manually coded data relative to models such as RoBERTa (Chae & Davidson, 2023).

We did find that zero-shot classification with recent GPT models delivered good performance when it came to detecting hate speech in tweets and identifying certain emotions in Reddit posts. In line with some recent work, this suggests that these models might be appropriate for certain tasks (Abdurahman et al., 2023; Rathje et al., 2023; Ziems et al., 2023). Still, on other tasks, the results of zero-shot classification with generative models need to be extensively validated (Kocoń et al., 2023; Ollion et al., 2023; Pangakis et al., 2023). Whereas we are optimistic and very excited about these new possibilities, given the current knowledge, we recommend using zero-shot classification with caution and validating the results against a reliable reference (Kristensen-McLachlan et al., 2023; Pangakis et al., 2023; Ziems et al., 2023).

Some issues, however, remain, particularly when using proprietary models for scientific research (Ollion et al., 2024). OpenAI, the company behind the GPT models, does not currently provide information on the full data used to train the latest models (Liesenfeld et al., 2023), nor does it enable their fine-tuning. Researchers cannot access the internal architecture of such proprietary models, which further limits our understanding of their capabilities and classification decisions. Additionally, specific versions of GPT models are quickly deprecated (usually within a year of their release), creating challenges for reproducibility. Recent research suggests using open-source alternatives such as Meta’s LLaMA, Stanford Alpaca, Mistral, or BLOOM (Gao et al., 2023; Spirling, 2023; Touvron et al., 2023; Zheng et al., 2023) and only relying on proprietary models if the benefits of doing so are clear (Palmer et al., 2023).

### Complexity-performance trade-offs

While researchers can begin to unravel the coding decisions of fine-tuned transformer models using specialized tools (Kokhlikyan et al., 2020; Lundberg & Lee, 2017), this is more challenging than understanding the decisions of dictionaries and simpler supervised machine learning methods. In addition, transformer models have other shortcomings, including performance variability (Mosbach et al., 2021) and reliance on “shallow” shortcuts for classification (Merchant et al., 2020). In some of our applications, the use of transformer models leads to relatively small improvements in performance compared to simpler methods. Moreover, despite their complexity, supervised models, in general, struggled to identify infrequently occurring categories. Our results suggest that simple custom-made dictionaries fare better on this task and could be used to supplement more complex methods in some cases.

### Working with complex coding schemes

Overall, we find that transformer classification models can tackle coding schemes of varying complexity well. In line with some recent research, we do find that it can be beneficial to use supervised machine learning models designed for category co-occurrence when working with particularly complex coding schemes (Erlich et al., 2022). Other methods, including dictionaries, logistic regression, and even zero-shot classification, tend to capture co-occurrence patterns less well.

### What is the best way to prepare textual data?

We find that stop word removal improved the performance of custom-made dictionaries, but lemmatization seemed to matter to a lesser extent. Text preparation when using “bag-of-words” representations with supervised machine learning models appeared to have a rather modest effect on model performance. Finally, we did not see any improvement upon using fine-tuned transformer models with pre-processing pipelines designed for short social media texts in particular (BERTweet). Our results, thus, echo the notion that there is no universally “best” approach to text pre-processing (Denny & Spirling, 2018), but also suggest that method choice matters more than specific pre-processing decisions taken when working with a particular method.

Before concluding, we emphasize that it is crucial to bear in mind the limitations of measuring concepts in text more generally. Although there is evidence that internal experiences are reflected in language use (Boyd & Pennebaker, 2017; Tausczik & Pennebaker, 2010) and can be recovered by external observers (Koutsoumpis et al., 2022), there are still important factors to consider. First, not all internal states appear to be equally observable in text (Kennedy et al., 2021). Second, it is still an open question to what extent manual coding for internal states accurately captures the intentions of the author, rather than manual coders’ subjective perceptions (Boyd & Schwartz, 2021; Kennedy et al., 2022; Koutsoumpis et al., 2022; Vazire, 2010). There is still a need for extensive validation of the concepts extracted from text by manual coders against alternative measures including self-reports, observer reports, physical states, and actual behaviors (Amador Diaz Lopez et al., 2017; Bleidorn & Hopwood, 2019; Boyd & Schwartz, 2021; Kennedy et al., 2021; Lykousas et al., 2019; Malko et al., 2021; Matsuo et al., 2019; Troiano et al., 2019; Vine et al., 2020). Finally, existing methods for measurement in text usually involve substantial simplification. For instance, nuanced internal states are often reduced to binary indicators (e.g., whether the emotion of joy is present in the text or not). While such reductions facilitate our analyses (including those relying on text mining), they can dispose of important details about the intensity with which individuals perceive and communicate their internal experiences.

Bearing these limitations in mind, we demonstrate how manual coding of internal states can be reliably extended across many texts using different text mining methods. While we examined method families separately for simplicity and clarity, researchers can also effectively combine them. For example, dictionaries can be enhanced with the help of distributed representations (Di Natale & Garcia, 2023; Garten et al., 2018; Mpouli et al., 2020), and dictionary outputs can serve as input for supervised machine learning methods (Farnadi et al., 2021). We consider text mining methods as valuable tools that can augment our analytical capabilities, but are prone to replicating the shortcomings of our research procedures (Grimmer et al., 2022). Therefore, it is important to rely on text-based measures that are rooted in theory and substantive knowledge about the concepts of interest (Grimmer et al., 2022; Kennedy et al., 2022), evolve through engagement with textual data (Lazer et al., 2021; Nelson, 2017), and are built with consideration of potential structural biases inherent in our data and computational methods (Bonikowski & Nelson, 2022).

Big textual data come with their own limitations for applications in social and behavioral sciences (Boyd & Schwartz, 2021; Kern et al., 2016; Lazer et al., 2021; Macanovic, 2022). Therefore, it could be beneficial to supplement them with questionnaire, census, or experimental data (Boyd & Schwartz, 2021; Salganik, 2017; van Loon et al., 2020). Finally, as we have done in this survey, it is crucial to conduct transparent and reproducible text mining analyses (Nelson, 2019) and validate model performance against a reliable (human) reference (Bonikowski & Nelson, 2022; Grimmer et al., 2022; Grimmer & Stewart, 2013; van Atteveldt et al., 2021). We conclude by reiterating the advice of Grimmer and colleagues (2021): despite the abundance of data and computational tools, we should not disregard the valuable lessons learned working with scarce data by prioritizing theory, sound research design, and good implementation.

## Footnotes

1 Text mining is a broad term denoting computer-assisted analysis of texts using techniques from natural language processing and machine learning (Hotho et al., 2005). We use this term interchangeably with “computational” or “automatic” text analysis.

2 Recent work by Bonikowski and Nelson (2022) and Hurtado Bodell and colleagues (2022) outlines some important considerations regarding these choices. The main unit of analysis can be whole texts, sentences, or segments of text (Barberá et al., 2021; Do et al., 2022).

3 Some methods do not require manually coded input to perform automatic coding, but should still be validated against a gold standard evaluated by humans (Grimmer & Stewart, 2013).

4 We discuss intercoder agreement measures in Appendix A. All appendices can be found at: https://osf.io/h2q5k.

5 Equations 1–3 in Appendix B provide more details on how these measures are calculated.

6 Since our implementations involve schemes where a text can be coded for multiple categories simultaneously (see Sect. "Case studies"), we fit separate logistic regression models for each category. In one implementation where a text can belong to only one of several categories, we fit a multinomial logistic regression.

7 We train separate RF and SVM models for each coding category of interest in applications where categories can co-occur across texts. For a comprehensive overview of other solutions for tackling co-occurrence with SML algorithms, see Erlich and colleagues (2022).

8 We use this term frequency (TF) approach in main analyses. Alternatively, a DTM can be normalized by the relative frequency of word occurrences in all texts (Aggarwal, 2018). We discuss this TF-IDF (term frequency-inverse document frequency) representation in Appendix C and show the results in Figures E5 and E6 in Appendix E. The unit being counted in a DTM does not have to be a word (or even any token) itself. For instance, one could represent texts as vectors of word counts belonging to certain dictionary categories.

9 In Appendix C, we describe our evaluations of SML models trained on such word representations from word2vec and GloVe models. We show the results in Figures E5 and E6 in Appendix E.

10 We use “*bert-base-uncased,*” “*roberta-base*,” “*bertweet-base*,” “*gpt-3.5-turbo-16k*,” and “*gpt-4*” models. Also see Appendix B.

11 Lemmatization replaces different word forms with their dictionary forms (e.g., “caring” will result in “care”). A similar procedure called stemming removes word suffixes (e.g., “caring” will result in “car”).

12 In one of our applications where a text can belong to one of the multiple categories, we classify it into the category it belongs to with the highest probability.

13 This approach has been shown to perform better than the computationally less intense procedure of fine-tuning only the upper model layers and the classification head (Lee et al., 2019).

14 During fine-tuning, the model processes texts in batches and updates parameters after each batch, completing an epoch once all training data are processed. We use a batch size of 8, with default values for other parameters (Devlin et al., 2019; Mosbach et al., 2021). See Appendix E and Figure E7 for a brief discussion on the impact of batch size and number of epochs on model performance on our tasks.

15 For example, if only one out of 100 texts should be coded for a certain category, a method not coding any texts for it would already achieve almost perfect accuracy of 0.99.

16 This measure is calculated by computing precision and recall across all categories and calculating the harmonic mean of the two. As such, it is less sensitive to poor performance on infrequently occurring categories. Micro-averaged *F*-score is equal to accuracy in applications where texts can belong to only one category at a time. In Figure D1 in Appendix D, we report several alternative measures.

17 In Figure E6 in Appendix E, we discuss the effects of varying these parameters in more detail.

18 However, as we show in Figures F6 and F7 in Appendix F, Dictionary 3 is more sensitive to the choice of a particular word cutoff criterion for the %DIFF measure. We suggest that researchers explore different cutoff parameters and manually screen included words when implementing our CMD creation procedure with %DIFF.
